# Changes in Sleep Problems and Psychological Flexibility following Interdisciplinary Acceptance and Commitment Therapy for Chronic Pain: An Observational Cohort Study

**DOI:** 10.3389/fpsyg.2016.01326

**Published:** 2016-08-31

**Authors:** Aisling Daly-Eichenhardt, Whitney Scott, Matthew Howard-Jones, Thaleia Nicolaou, Lance M. McCracken

**Affiliations:** ^1^INPUT Pain Management, Guys and St. Thomas NHS Foundation Trust HospitalsLondon, UK; ^2^Department of Psychology, Institute of Psychiatry, Psychology, and Neuroscience, King's College LondonLondon, UK

**Keywords:** chronic pain, insomnia, acceptance and commitment therapy

## Abstract

**Aims:** Cognitive and behavioral treatments (CBT) for sleep problems and chronic pain have shown good results, although these results could improve. More recent developments based on the psychological flexibility model, the model underlying Acceptance and Commitment Therapy (ACT) may offer a useful addition to traditional CBT. The aim of this study was to examine whether an ACT-based treatment for chronic pain is associated with improved sleep. Secondly, we examined the associations between changes on measures of psychological flexibility and sleep-related outcomes.

**Methods:** The study used an observational cohort methodology. Participants were 252 patients (73.8% female) attending a 4-week, interdisciplinary, pain management program in London, United Kingdom. Participants completed standard self-report measures of pain and functioning, sleep outcomes, and processes of psychological flexibility. Pre- to post-treatment, and pre-treatment to follow-up measures were examined for statistically significant differences using paired samples *t*-tests. Secondarily, hierarchical multiple regression analyses were conducted to examine change in process measures in relation to change in treatment outcome.

**Results:** Participants showed statistically significant improvements (all *p* < 0.001) at post-treatment on measures of insomnia severity (*d* = 0.45), sleep interference (*d* = 0.61), and sleep efficiency (*d* = 0.32). Significant improvements in insomnia severity and sleep interference were also observed at 9-month follow up. Small to medium effect sizes were observed across the sleep outcomes. Statistically significant changes were also observed on measures of psychological flexibility, and these improvements were significantly associated with improvements on sleep-related outcomes, independently contributing up to 19% of unique variance.

**Conclusion:** This study supports the potential usefulness of ACT-based treatments for chronic pain for addressing co-occurring sleep difficulties. Further research is needed to determine how to improve the impact of this treatment for co-morbid pain and sleep difficulties, possibly using a randomized-controlled trial design.

## Introduction

Sleep is clearly an important factor in the experience of chronic pain. When people with chronic pain are asked to identify the most important areas of their life impacted by pain, sleep is rated among their top five (Turk et al., 2008). The prevalence of insomnia in people with chronic pain is at least twice as high as in those without chronic pain (Silverstein et al., 2009). In people seeking treatment for chronic pain, 53% in secondary care (Tang et al., 2007) and 79% in tertiary care (McCracken et al., 2011) screen positive for significant insomnia. Pain appears to contribute to sleep disorders (Fishbain et al., 2010) and poor sleep appears to increase pain and emotional distress (Haack and Mullington, 2005; Bonvanie et al., 2016). There is likely a bi-directional relationship between chronic pain and insomnia (Koffel et al., 2016). Given that both chronic pain and sleep difficulties are independently linked to reduced quality of life, a greater focus on addressing insomnia in the context of chronic pain is needed (Currie et al., 2002; Smith and Haythornthwaite, 2004; Tang et al., 2007; Fishbain et al., 2010; McCracken et al., 2011).

Cognitive and behavioral treatments (CBT) for insomnia appear to produce significant and lasting improvements in sleep (e.g., Morin et al., 2006). Thus, CBT presents a natural opportunity for addressing insomnia in the context of chronic pain. An earlier review of 13 longitudinal studies of CBT related to insomnia and chronic pain, suggested that CBT may offer benefits for reducing pain and improving sleep (Smith and Haythornthwaite, 2004). An early study of CBT for insomnia in people with chronic pain showed 57% of participants achieved a reliable improvement, although only 18% fully recovered from sleep problems (Currie et al., 2002). In another pilot study, CBT designed to address both pain and insomnia in people with chronic pain appeared feasible and possibly superior to CBT for either pain or insomnia alone, but only in terms of sleep outcomes (Pigeon et al., 2012). A much larger trial of combined CBT for pain and insomnia (*N* = 367 older adults with osteoarthritis) similarly showed favorable outcomes for insomnia severity but not for pain (Vitiello et al., 2013). In a more recent pilot trial of a “cognitive behavioral pain management program” compared to a waiting list condition the former produced better results for anxiety, depression, and kinesiophobia; however, on most of the sleep measures it did not produce a better result (Blake et al., 2015). In analyses of long terms effects of combined CBT for pain and insomnia there were no differences between combined CBT, CBT for pain alone, or an education only control condition at 18-month follow-up in older adults with osteoarthritis (McCurry et al., 2014). Only in an *ad hoc* analysis of selected participants with severe pain and insomnia did an effect of the combined treatment emerge, and only for pain severity. Hence, there appears to be a lack of reliable effects in trials of CBT for chronic pain or insomnia, or both, when considering both measures of insomnia and other pain-related outcomes.

A newer generation of CBT may improve the ways we treat the collateral problems of chronic pain and insomnia. These treatments include processes of mindfulness and acceptance (Ong et al., 2008, 2012) or a related broader process, psychological flexibility (McCracken et al., 2011; Hayes et al., 2012). Psychological flexibility includes a set of behavioral capacities, including acceptance, present moment awareness, and goal-directed or values-based activation skills. The current treatment approach most specifically focused on increasing psychological flexibility is Acceptance and Commitment Therapy (ACT; Hayes et al., 2012). ACT is a form of CBT that includes experiential, exposure-based, awareness-focused, and activation and motivation focused methods directed toward building behavior that is “open, aware, and active,” the essential qualities of psychological flexibility (Hayes et al., 2011).

Results from previous cross-sectional studies show that acceptance, mindfulness, and values-based action correlate significantly with measures of insomnia severity, and daytime rest (McCracken et al., 2011; Bothelius et al., 2015). The benefits for sleep from acceptance, mindfulness, and values-based action processes may derive from the ways they coordinate less struggling with feelings, less entanglement in arousing and distressing thoughts, and less daytime rest and disengagement, thus facilitating improved patterns of nighttime sleep and daytime activity (McCracken et al., 2011). While there has been published treatment development work focusing on adding mindfulness methods to CBT for insomnia (Ong et al., 2008), as far as we are aware there is not yet a published prospective or treatment outcome study of psychological flexibility and insomnia or sleep outcomes in people with chronic pain. Such a study would be a next logical step for exploring the potential role of this set of “newer generation” processes in this area.

The primary purpose of the current study was to investigate changes in insomnia and sleep-related difficulties following interdisciplinary treatment for chronic pain. A secondary purpose was to examine associations between changes in processes of psychological flexibility and changes in sleep outcomes following treatment. A relatively large cohort of participants in a service for complex chronic pain problems received treatment based on ACT. Patients received two formal sessions aimed at addressing sleep problems—the specific treatment content aimed at improving sleep was therefore minimal. Standard pain treatment outcomes as well as insomnia severity, sleep interference, estimated sleep efficiency, and sleep medication use were assessed before and after treatment and at a 9 month follow-up. At the same assessment intervals, facets of psychological flexibility, including pain acceptance, cognitive fusion, decentering, and committed action, were also assessed. It was predicted that both standard pain and functioning outcomes and sleep-related outcomes would improve significantly. In the secondary analyses, it was predicted that psychological flexibility processes would improve significantly, and that changes in psychological flexibility would account for significant variance in improvements in sleep outcomes.

## Methods

### Participants and procedures

Participants were recruited from 299 consecutive patients attending a 4-week, interdisciplinary, pain management program between August 2014 and September 2015 in London, United Kingdom. Patients were screened and selected for treatment by a physiotherapist and a psychologist using the following criteria: pain lasting more than 6 months that significantly impacted on day to day living. Patients were excluded if they had any poorly controlled psychiatric condition or neurocognitive impairment that might interfere with treatment or if they were unwilling to attend a group-based treatment program. A semi-structured interview was carried out by a psychologist and a physiotherapist to assess pain-related distress and disability. The physiotherapist also conducted a physical examination or performance tests as needed. These methods are the routine assessment and selection process within the service. Selection for treatment was at the discretion of the assessing clinicians on a case-by-case basis, rather than on the basis of scores on standardized measures of distress and disability.

A total of 299 patients began treatment. Thirteen patients did not consent to have their data used for research purposes and, therefore, were excluded from the analyses. A further 28 people (9.4%) did not complete treatment and were likewise excluded from the current study. Another six people completed treatment, but did not complete post-treatment questionnaires. Therefore, the final sample included in the pre- to post-treatment analyses consisted of 252 participants. Of these 252 people, 153 (61%) returned for their 9 month follow-up assessment and completed questionnaires. Therefore, follow-up data analyses were computed on this subsample of 153 people.

Participants completed a standard baseline assessment on the first day of treatment, during which they reported their sex, age, ethnicity, pain location and duration, living situation, and employment status. The pre-treatment assessment also included measures of pain intensity, pain interference, depression, insomnia severity, sleep efficiency, sleep interference, and measures of processes of psychological flexibility. Participants completed the same measures during the final week of treatment and at a 9 month follow-up assessment. Use of hypnotic and anxiolytic medications, as categorized by the British National Formulary (BNF; Royal Pharmaceutical Society of Great Britain, 2011), was recorded by a nurse on the first day and the final week of treatment. Hypnotic and anxiolytic medications were selected for analysis as these are the drug groups currently recommended for management of insomnia in the UK (NICE, 2015). These data were gathered using prescription records and self-report, and recorded in the database as taking or not taking. The research database and study were granted ethics and National Health Service Research and Development approvals prior to commencing data collection.

### Measures

#### Pain intensity

Participants rated their pain intensity on average over the last week on a standard scale from 0 (no pain) to 10 (extremely intense pain).

#### Pain interference

The Brief Pain Inventory (BPI) is a measure of pain severity and interference (Cleeland and Ryan, 1994). In the current study we used the seven-item interference scale from this measure. The interference scale includes items related to general activity, mood, walking, work, relations with others, sleep and enjoyment of life, each rated with regard to how much pain interferes, from 0 (does not interfere) to 10 (completely interferes). The interference score is calculated as a mean of the seven interference item ratings. For the purpose of this study, the sleep interference item was also used as an individual rating. The BPI is widely used and recommended in consensus guidelines as a measure of pain clinical trials (Dworkin et al., 2005). The BPI demonstrated good internal consistency in the current sample (Cronbach's α = 0.85).

#### Insomnia severity

The Insomnia Severity Index (ISI; Bastien et al., 2001) is a seven item screening measure of insomnia severity. Participants are asked to consider the last 2 weeks and rate the severity of their difficulties falling asleep, staying asleep, sleep quality and its impact on daily functioning, as well as their concerns on a scale of 0 (not at all) to 4 (extremely). Higher scores indicate more severe sleep problems. Items are summed to produce a total score, with higher scores reflecting greater severity of insomnia. Total scores are categorized as not clinically significant (0–7), sub threshold (8–14), moderate insomnia (15–21), and severe insomnia (22–28; Morin et al., 2011). The ISI has been validated and shows good internal consistency in clinical samples (Cronbach's α = 0.74) (Bastien et al., 2001) including in the current sample (Cronbach's α = 0.87).

#### Sleep efficiency

Participants reported on their total time spent in bed and total sleep time on a “typical night in the past 2 weeks.” A sleep efficiency rating was calculated by multiplying the ratio of total sleep time to total time spent in bed by 100 (McCracken et al., 2011).

#### Depression

The Patient Health Questionnaire (PHQ-9, Kroenke et al., 2001) was used to measure depression. Participants rated how frequently they experienced nine common symptoms of depression in the last 2 weeks using a scale of 0 (not at all) to 3 (nearly every day). Higher total scores indicate greater severity. The measure has been well validated among people with chronic health conditions (Kroenke et al., 2001) and demonstrated good internal consistency in the current sample (Cronbach's α = 0.83).

#### Pain acceptance

The eight-item Chronic Pain Acceptance Questionnaire (CPAQ-8) was used to measure acceptance of chronic pain. It reflects the engagement in normal daily activities with pain and cessation of ineffective avoidance or control strategies (McCracken et al., 2004; Fish et al., 2010). Each item is rated on a scale of 0 (never true) to 6 (always true) and higher total scores indicate greater acceptance of pain. The CPAQ-8 has been validated and shown to have good reliability in people with chronic pain (Fish et al., 2010). The CPAQ-8 showed acceptable internal consistency in the current sample (Cronbach's α = 0.71).

#### Cognitive fusion

Cognitive fusion, the failure to experience a distinction between the content of thoughts and direct experience, was measured using the self-report seven item measure, the Cognitive Fusion Questionnaire (CFQ-7; Gillanders et al., 2014). Cognitive defusion in contrast, is similar to mindfulness processes in which participants see their thoughts as transient events that may or may not reflect reality, with the aim of reducing their impact. Participants are asked to rate how true a list of statements are for them using a scale of 1 (never true) to 7 (always true). When summed, higher total scores indicate greater cognitive fusion. The CFQ-7 has previously been validated among people with chronic pain (McCracken et al., 2013a) and demonstrated excellent internal consistency in the current sample (Cronbach's α = 0.95).

#### Decentering

The 12-item decentering scale from the Experiences Questionnaire (EQ; Fresco et al., 2007; McCracken et al., 2013b) was used here. It reflects the ability to observe one's thoughts and feelings as temporary, objective events in the mind and not necessarily true reflections of oneself or one's circumstances. Each statement is rated on a scale of 1 (never) to 5 (always). Higher total scores suggest greater decentering. The EQ has been validated among people with chronic pain (McCracken et al., 2013b). The decentering scale showed good internal consistency in the current sample (Cronbach's α = 0.85).

#### Committed action

The Committed Action Questionnaire (CAQ-8; McCracken, 2013; McCracken et al., 2015) was used to measure flexible, goal-oriented behavior. The measure consists of eight items and asks participants to rate how true a list of statements are for them, using a scale of 0 (never true) to 6 (always true). The item pool consists of four positively and four negatively phrased items. Negatively phrased items are reverse scored before the total score is calculated, with higher scores indicating greater committed action. The reliability and validity of the CAQ is supported by previous research in a chronic pain population (McCracken et al., 2015) and this good internal consistency was evident in the current sample (Cronbach's α = 0.83).

### Treatment program

The treatment here used principles and methods of ACT within a multidisciplinary rehabilitation context comprised of psychologists, occupational therapists, physiotherapists, nurses, and physicians. Treatment was provided in a group format, of up to 12 participants, for four days a week over four weeks. All professionals had received extensive training in and routinely worked within an ACT model, and attended continuing professional development training to facilitate further improvement in treatment delivery. As part of routine clinical practice, regular team meetings, and clinical development sessions were held to ensure treatment fidelity and promote clinical competency. Participants were expected to attend all sessions, although attendance data are not available. Treatment sessions lasted one hour on average and were divided among all professions, with the largest proportion including psychology, physiotherapy, and occupational therapy. Treatment sessions were designed to develop key processes of psychological flexibility: openness to experiencing pain and unwanted feelings; present moment awareness; and values-guided behavior. Pain reduction and controlling unwanted thoughts and feelings were not an explicit focus of treatment. Instead, the emphasis of treatment was on experiential exercises, use of metaphor, mindfulness practice, cognitive defusion techniques, and values-based methods in order to promote improved daily functioning and general wellbeing. These were used across disciplines in addition to goal-setting and educational approaches.

The sleep component of the treatment was based on a cognitive behavioral approach for treating insomnia and was routinely delivered by an occupational therapist. The therapist had the relevant clinical experience and had a particular interest in this area. Occupational therapists are well placed to deliver interventions that are empowering and facilitate behavioral change. The sleep component was delivered over two, hour-long, group sessions in the second and third week of the treatment program. The two group-based sessions were followed up with individual sessions, if necessary.

The first group-based sleep session focused on exploring beliefs the patients held about their sleep and provided information to assist them to reframe their experience of sleeplessness. Patients were asked to keep a 7-day sleep diary to examine their current pattern of sleep and their perception of quality of sleep (Carney et al., 2012). The second group-based sleep session incorporated practice of ACT-based techniques covered in the treatment program and relating them to struggles with sleep, such as defusion from insomnia-related thoughts and mindfulness skills. Participants were invited to practice the psychological flexibility skills in relation to sleep problems during the evenings both in the residential setting and at home. This was reviewed in the structure of the sleep session during the 4 weeks and also at the 1 and 9 month follow-up session. The completed sleep diary was used to highlight individuals who could benefit from individual sessions focusing on scheduling a new sleep pattern. Patients were invited to attend two individual sessions if the diary highlighted a particularly poor sleep routine or poor sleep efficiency, for example 10 h in bed and 5 h asleep = 50% efficient.

The individual work focused on a sleep compression approach with the aim of establishing an improved sleep pattern and efficiency (Espie, 2012). The completed sleep diary was used to establish an average amount of time spent in bed compared to time spent sleeping. A new sleep pattern was planned with the getting up time remaining the same, getting into bed at a later time and the amount of time in bed reduced to a minimum of 6 h. This usually involved two individual sessions up to 1 h each with progress reviewed at a 1 month follow up. Again, skills covered in the 4 week treatment course were also applied to struggles related to sleep. The number of patients who received 1:1 session was not recorded.

### Statistical analyses

Data analyses were conducted using SPSS version 22. Means and standard deviations were computed for all measures for pre- and post-treatment and follow-up. Across the pre- and post-treatment assessments, the largest percentage of missing data attributable to a single item within a given questionnaire was 1.2%. At the follow-up assessment, the largest percentage of missing responses attributable to a single item on a questionnaire was 2.6%. Therefore, missing item-level data were considered to be missing completely at random. For questionnaires with 7 or more items (i.e., the BPI, ISI, PHQ-9, CPAQ-8, CFQ-7, EQ, and CAQ-8), person mean substitution was used to impute missing values for participants missing only a single item. Scores were not imputed for participants missing two or more items on these questionnaires, or for individual assessment items (i.e., pain intensity, sleep interference, estimated sleep time, and total time in bed). Following imputation, the range of participants with missing data at pre- or post-treatment on the questionnaire total scores was 0–4%. At follow-up, there was a range of 0–5% missing data across the questionnaire total scores.

Medication use was categorized as the proportion of patients taking hypnotic and anxiolytic medication (yes/no) at pre- and post-treatment. Participants' total scores on the ISI were categorized according to previously established clinical cut-offs for severity at pre-, post-treatment, and follow-up. Sleep efficiency was calculated as participants' estimated sleep time divided by their total reported time in bed, and multiplied by 100. Sleep efficiency was not computed for participants with missing data on either (or both) estimated sleep time or total time in bed. Since a sleep efficiency of greater than 100 percent is not interpretable, participants for whom sleep efficiency scores were >100 were removed from this analysis (number of cases removed was six, four, and one for pre-treatment, post-treatment, and follow-up, respectively). Independent samples *t*-tests and Chi-square tests were computed to examine differences on pre-treatment assessment and demographic variables for treatment and follow-up completers and non-completers.

The clinical significance of changes for the following treatment outcome variables were also examined: pain intensity, pain interference, insomnia severity, sleep interference, sleep efficiency, and depression. For these analyses, raw change scores greater than one half of a standard deviation from baseline score for each respective outcome variable were coded as “clinically improved” (Norman et al., 2003). All participants whose scores did not improve by one half of a standard deviation were coded as “not clinically improved,” while those who worsened by greater than half of a standard deviation were coded as “clinically worsened.” Frequencies were tabulated for the proportion of individuals with change scores in each of these categories.

A series of paired-samples *t*-tests were computed to examine differences on assessment measures between pre- to post-treatment and pre-treatment to follow-up. Normality was assessed through examination of skewness and kurtosis values (between −2 and +2), and inspection of histograms and normal q-q plots. For the pre- to post-treatment analyses, all of the paired differences were considered to be normally distributed except for sleep efficiency. For the pre- to follow-up analyses, the paired differences were considered to be normally distributed for all variables except sleep efficiency. Cases with raw change scores that were more than three standard deviations from the mean change in either direction were considered as outliers and removed from the analysis. Following this procedure, 2 cases were removed for the pre- to post-treatment, and pre- to follow-up paired comparisons for sleep efficiency. Following removal of these outliers, the paired differences for these variables were considered to be normally distributed. Within-subjects effect sizes (Cohen's d) were calculated as the difference between pre- and post-treatment means, and pre-treatment and follow-up means divided by the pooled standard deviation. Effect sizes were interpreted as small (>0.20), medium (>0.50), or large (>0.80) in accordance with Cohen's guidelines (Cohen, 1992). McNemar tests were used to compare the proportion of participants who were and were not taking hypnotic and anxiolytic medications before and after treatment.

A correlation analysis of changes on psychological flexibility processes and changes in treatment outcomes was calculated using residualized change scores. Residualized change scores were used rather than raw change scores as they account for the influence of baseline scores on subsequent assessment values, whereas raw change scores do not. Residualized change scores were computed using the baseline score of a variable to predict the post-treatment or follow-up value of the variable in a regression analysis; the residualized change score was computed as the difference between the predicted and actual post-treatment or follow-up score with the baseline covaried out. Pearson correlations using residualized change scores were then computed to examine the relationship between changes on psychological flexibility processes and treatment outcomes.

Hierarchical multiple regression analyses were computed to examine the shared and unique contributions of change in psychological flexibility variables to change in sleep outcomes from pre- to post-treatment and pre-treatment to follow-up. Changes in pain intensity were controlled for in the first step of each regression analysis. Psychological flexibility processes (i.e., pain acceptance, cognitive fusion, decentering, and committed action) were entered in the second step of the regression equations. To maximize sample size for all analyses, pairwise deletion was used to address missing values on study variables. Therefore, the sample size varies slightly across the *t*-tests, correlations, and regression analyses, depending on the variables being examined. Degrees of freedom and sample sizes are reported throughout the analyses to reflect these minor differences.

## Results

### Sample demographics

The majority of the sample was female (73.8%) and white European (74.0%). The sample had an average age of 45.3 years (SD = 12.2), and median pain duration of 102.0 months (IQR = 164.0). The most frequent pain site was generalized pain (41.8%), followed by pain in the lower back (38.2%). The majority of the sample (53.2%) was unemployed at the time of assessment. Further demographic characteristics of the sample are presented in Table 1.

**Table 1:** **Characteristics of study sample**.

**Variable**	***n* (%) or *M* (SD)**
**GENDER**	
Male	66 (26.2)
Female	186 (73.8)
Age (years)	45.34 (12.24)
Pain duration (months)^*^	102.00 (164.00)
**MAIN PAIN SITE**	
Head	5 (2.0)
Neck	6 (2.4)
Upper limbs	9 (3.6)
Chest	2 (0.8)
Abdominal	5 (2.0)
Lower back	96 (38.2)
Lower limbs	21 (8.4)
Pelvic	2 (0.8)
Generalized	105 (41.8)
Missing	1 (0.4)
**ETHNIC GROUP**	
White	185 (74.0)
Black	28 (11.2)
Asian	17 (6.8)
Latin/Hispanic	6 (2.4)
Mixed	14 (5.6)
Missing	2 (0.8)
**LIVING STATUS**	
Alone	60 (23.8)
With partner	57 (22.6)
With child/children	34 (13.5)
With partner and child/children	72 (28.6)
With other relatives	22 (8.7)
With friends/flatmates	7 (2.8)
**EMPLOYMENT STATUS**	
Full-time	37 (14.7)
Part-time	27 (10.7)
Unemployed	134 (53.2)
Volunteer	10 (4.0)
Student	6 (2.4)
Homemaker/Carer	16 (6.3)
Retired	21 (8.3)

* *Pain duration reported as median and interquartile range*.

People who completed more years of education (*M* = 14.0; *SD* = 4.0) were more likely to complete treatment than those who completed less (*M* = 12.5; *SD* = 3.4), *t*_(281)_ = 2.03, *p* < 0.05. Treatment completers and non-completers did not differ significantly in terms of other demographic variables or any pre-treatment assessment variable. Compared to follow-up non-completers, follow-up completers scored significantly higher on chronic pain acceptance at post-treatment (*M* = 24.61; SD = 7.38 vs. *M* = 21.62; SD = 7.85), *t*_(248)_ = 3.04, *p* < 0.01, and on committed action at post-treatment (*M* = 28.72; SD = 6.84 vs. *M* = 26.71; SD = 7.32), *t*_(250)_ = 2.22, *p* < 0.05. Participants who did and did not complete the follow-up assessment did not differ significantly on any other post-treatment assessment or demographic variables.

At the beginning of treatment, the sample reported spending an average of 9.01 h (SD = 3.31) in bed between going to bed at night and getting up in the morning. The average estimated sleep time of the sample was 5.30 h (SD = 2.27). Based on established clinical cut-offs for interpreting the ISI, ~81.3% of the sample scored in the clinically significant range (i.e., moderate or severe) in terms of the severity of their insomnia symptoms (Morin et al., 2011).

### Treatment changes on sleep and psychological flexibility variables

Table 2 summarizes descriptive statistics for scores on pain intensity and the sleep and psychological flexibility measures at pre- and post-treatment and follow-up. Paired-samples *t*-tests indicated that pain intensity, pain interference, depression, insomnia severity, sleep interference, and sleep efficiency showed significant improvements from pre- to post-treatment. Large effect sizes were observed for improvements in pain interference and depression. A medium effect size was observed on pain intensity and sleep interference. Small effect sizes were seen for insomnia severity and sleep efficiency. Each of the psychological flexibility variables likewise showed statistically significant improvements from pre- to post-treatment. The magnitude of changes in cognitive fusion and committed action were near small and small, while the changes for decentering and pain acceptance were medium and large, respectively. At the end of treatment 67.0% of participants met criteria for clinically significant insomnia on the ISI, compared to 81.3% before treatment (Table 3).

**Table 2:** **Changes in sleep and psychological flexibility process variables**.

	**Pre-treatment**	**Post-treatment**	***t (df)***	***d***	**Follow-up**	***t (df)***	***d***
Pain intensity	7.84 (1.65)	6.62 (1.73)	*t*_(249)_ = 10.78^***^	0.72	7.33 (1.94)	*t*_(150)_ = 3.08^**^	0.25
Pain Interference (Average)	7.81 (1.51)	5.85 (2.04)	*t*_(247)_ = 15.16^***^	1.09	6.60 (2.17)	*t*_(144)_ = 6.66^***^	0.62
Depression	17.50 (5.53)	11.71 (5.71)	*t*_(243)_ = 16.41^***^	1.03	13.95 (6.49)	*t*_(145)_ = 7.22^***^	0.60
Insomnia severity	20.03 (5.82)	17.20 (6.75)	*t*_(249)_ = 7.62^***^	0.45	17.81 (6.20)	*t*_(145)_ = 4.68^***^	0.36
Sleep Interference	8.05 (2.21)	6.53 (2.73)	*t*_(251)_ = 9.36^***^	0.61	7.25 (2.59)	*t*_(152)_ = 3.83^***^	0.33
Sleep efficiency	60.03(19.84)	66.30 (19.60)	*t*_(228)_ = −4.72^***^	0.32	60.74(19.87)	*t*_(138)_ = −0.47	0.04
Pain acceptance	17.22 (7.54)	23.44 (7.70)	*t*_(248)_ = −12.09^***^	0.81	24.55 (8.10)	*t*_(148)_ = −10.95^***^	0.91
Cognitive fusion	30.41 (11.26)	28.54 (10.54)	*t*_(247)_ = 3.23^***^	0.17	25.01 (10.64)	*t*_(143)_ = 5.62^***^	0.43
Decentering	35.39 (7.69)	39.41 (7.48)	*t*_(241)_ = −7.60^***^	0.53	39.52 (7.90)	*t*_(141)_ = −5.48^***^	0.52
Committed action	26.30 (8.47)	27.96 (7.08)	*t*_(246)_ = −3.42^***^	0.21	28.78 (7.37)	*t*_(143)_ = −2.02^*^	0.17

*** p ≤ 0.001;

** p < 0.01;

* *p < 0.05*.

**Table 3:** **Proportion of participants with clinically meaningful scores on the Insomnia Severity Index at pre- and post-treatment and follow-up**.

**Insomnia severity category**	**Pre-treatment *n* (%)**	**Post-treatment *n* (%)**	**Follow-up *n* (%)**
Not clinically significant	7 (2.8)	24 (9.5)	7 (4.8)
Sub-threshold	40 (15.9)	59 (23.4)	34 (23.1)
Clinically significant: Moderate	86 (34.1)	89 (35.3)	58 (39.5)
Clinically significant: Severe	119 (47.2)	80 (31.7)	48 (32.7)
Missing	0 (0)	0 (0)	6 (3.9)

*ISI, Insomnia Severity Index; Not clinically significant, ISI = 0–7; Sub-threshold, ISI = 8–14; Moderate, ISI = 15–21; Severe, ISI = 22–28. Pre- and post-treatment N = 252; Follow-up N = 147*.

Pre- and post-treatment hypnotic and anxiolytic medication use was recorded for 249 participants. At the beginning of treatment 22 (8.8%) participants were taking 1 or more hypnotic medication, while 227 (91.2%) of participants were taking none. At post-treatment, 17 (6.9%) were taking hypnotic medication, while 229 (93.1%) were not. A McNemar's test revealed that this difference in proportions was not statistically significant *p* = 0.06. At the beginning of treatment 48 (19.3%) participants were taking 1 or more anxiolytic medication, while 201 participants (80.7%) of participants were not. At post-treatment, 38 (15.4%) were taking anxiolytic medication, while 208 (84.6%) were not. A McNemar's test revealed that this difference in proportions was statistically significant *p* = 0.006.

From pre-treatment to follow-up, significant improvements were seen for all of the variables with the exception of sleep efficiency. Pain acceptance showed a large effect size from pre-treatment to follow-up. Effect sizes were medium for follow-up changes in pain interference, depression, and decentering. The follow-up changes in pain intensity, insomnia severity, sleep interference, cognitive fusion, and committed action were small to near small. At the 9 month follow-up, 72.2% of the sample met criteria for clinically meaningful insomnia.

Table 4 shows the proportion of patients reporting clinically significant improvements on outcome measures from pre- to post-treatment, and pre-treatment to follow-up. Across the outcome measures, 58.8% of patients showed clinically meaningful improvement from pre- to post-treatment on average. The proportion of patients reporting clinically meaningful improvements over this time period ranged from 42.7% for sleep efficiency to 84.5% for pain interference. At follow-up, an average of 47.3% of patients reported significant improvement compared to pre-treatment. The proportion of patients showing clinically meaningful improvements during this interval ranged from 28.8% for sleep efficiency to 69.0% for pain interference.

**Table 4:** **Clinically significant change on outcome variables**.

	**Pre- to post-treatment *n* (%)**			**Pre-treatment to follow-up *n* (%)**		
	**Significantly worse**	**No change**	**Significantly improved**	**Significantly worse**	**No change**	**Significantly improved**
Pain intensity	30 (12.0)	62 (24.8)	158 (63.2)	37 (24.5)	48 (31.8)	66 (43.7)
Pain interference	35 (13.9)	4 (1.6)	213 (84.5)	41 (28.3)	4 (2.8)	100 (69.0)
Depression	15 (6.1)	53 (21.7)	176 (72.1)	21 (14.4)	40 (27.4)	85 (58.2)
Insomnia severity	38 (15.2)	100 (40.0)	112 (44.8)	24 (16.4)	53 (36.3)	69 (47.3)
Sleep interference	16 (6.3)	122 (48.4)	114 (45.2)	17 (11.1)	80 (52.3)	56 (36.6)
Sleep efficiency	45 (19.8)	85 (37.4)	97 (42.7)	33 (23.7)	66 (47.5)	40 (28.8)

### Correlations between changes on psychological flexibility processes and sleep outcomes

Pearson correlations between residualized change scores for sleep outcomes and psychological flexibility variables are displayed in Table 5. Changes in the sleep outcome variables were all significantly inter-correlated. Improvements in pain intensity, pain interference, and depression were significantly correlated with improvements in insomnia severity and sleep interference. Improvements on all of the psychological flexibility processes were significantly correlated with improvements in insomnia severity and sleep interference. The same pattern of correlations was observed between changes in these variables from pre-treatment to follow-up. Pre- to post-treatment changes in sleep efficiency were significantly correlated with pre- to post-treatment changes in pain intensity, pain interference, depression, cognitive fusion, decentering, and committed action; changes in sleep efficiency at follow-up were significantly correlated with changes in pain interference, depression, and committed action.

**Table 5:** **Correlations between change in sleep and psychological flexibility process variables**.

	**Pre- to post-treatment change**			**Pre-treatment to follow-up change**		
	**Insomnia severity**	**Sleep interference**	**Sleep efficiency**	**Insomnia severity**	**Sleep interference**	**Sleep efficiency**
Pain intensity	0.34^***^	0.38^***^	−0.17^*^	0.37^***^	0.58^***^	−0.13
Pain Interference	0.57^**^	0.76^***^	−0.34^***^	0.45^***^	0.75^***^	−0.21*
Depression	0.58^***^	0.45^***^	−0.33^***^	0.57^***^	0.40^***^	−0.33^***^
Pain Acceptance	−0.37^***^	−0.30^***^	0.12	−0.34^***^	−0.51^***^	0.14
Cognitive Fusion	0.44^***^	0.26^***^	−0.20^**^	0.27^**^	0.16^*^	0.03
Decentering	−0.30^***^	−0.20^**^	0.20^**^	−0.25^**^	−0.25^**^	0.14
Committed Action	−0.31^***^	−0.16^*^	0.15^*^	−0.35^***^	−0.26^**^	0.20^*^

Pre- to post-treatment N = 227–252; Pre-treatment to follow-up N = 130–151;

*** *p ≤ 0.001*,

** *p ≤ 0.01*,

* *p ≤ 0.05*.

### Regression analyses examining contributions of change in psychological flexibility variables to change in sleep outcomes

Hierarchical multiple regression analyses were conducted to examine the shared and unique contributions of changes in psychological flexibility processes to improvements in sleep outcomes for the pre- to post-treatment interval (Table 6). Change in insomnia severity was the dependent variable for the first set of analyses. Change in pain was entered in the first step of this analysis, and significantly contributed 11% of the variance to change in insomnia severity. Changes in psychological flexibility processes (i.e., pain acceptance, cognitive fusion, decentering, and committed action) were entered in the second step and together contributed an additional 15% of the variance to change in insomnia severity, above and beyond that accounted for by changes in pain. Examination of the beta weights from the final regression equation indicated that changes in pain intensity, β = 0.22, *t*_(240)_ = 3.72, *p* < 0.001, pain acceptance, β = −0.18, *t*_(240)_ = 2.65, *p* < 0.01, and cognitive fusion, β = 0.24, *t*_(240)_ = 3.46, *p* < 0.001 each contributed significant unique variance to the prediction of change in insomnia severity. In the pre-treatment to follow-up regression analysis (Table 7), changes in psychological flexibility processes likewise significantly contributed an additional 13% of the variance to changes in insomnia severity, above and beyond the variance accounted for by changes in pain intensity (10%). For this follow-up analysis, changes in pain intensity and pain acceptance each contributed uniquely to changes in insomnia severity in the final regression equation, β = 0.24, *t*_(116)_ = 2.77, *p* < 0.01, and β = −0.25, *t*_(116)_ = −2.62, *p* = 0.01, respectively.

**Table 6:** **Regression analyses examining contributions of change in psychological flexibility processes to change in sleep variables from pre- to post-treatment**.

	**ΔR^2^**	***F*change *(df)***	***p***	**β**
**DV: INSOMNIA SEVERITY**				
Step 1	0.11	30.93 (1, 244)	<0.001	
Pain intensity				0.22^***^
Step 2	0.15	12.56 (4, 240)	<0.001	
Pain acceptance				−0.18^**^
Cognitive fusion				0.24^***^
Decentering				−0.02
Committed action				−0.07
**DV: SLEEP INTERFERENCE**				
Step 1	0.15	42.04 (1, 244)	<0.001	
Pain intensity				0.31^***^
Step 2	0.05	3.73 (4, 240)	<0.01	
Pain acceptance				−0.17^*^
Cognitive fusion				0.09
Decentering				−0.02
Committed action				0.01
**DV: SLEEP EFFICIENCY**				
Step 1	0.03	6.71 (1, 223)	=0.01	
Pain intensity				−0.13
Step 2	0.03	1.99 (4, 219)	*ns*	
Pain acceptance				−0.01
Cognitive fusion				−0.09
Decentering				0.12
Committed action				0.01

*** *p ≤ 0.001*,

** *p ≤ 0.01*,

* *p < 0.05*.

**Table 7:** **Regression analyses examining contributions of change in psychological flexibility processes to change in sleep variables from pre-treatment to follow-up**.

	**ΔR^2^**	***F*change *(df)***	***p***	**β**
**DV: INSOMNIA SEVERITY**				
Step 1	0.10	13.10 (1, 120)	<0.001	
Pain intensity				0.24^**^
Step 2	0.13	4.90 (4, 116)	=0.001	
Pain acceptance				−0.24^**^
Cognitive fusion				0.09
Decentering				0.06
Committed action				−0.16
**DV: SLEEP INTERFERENCE**				
Step 1	0.32	58.43 (1, 125)	<0.001	
Pain intensity				0.48^***^
Step 2	0.19	11.80 (4, 121)	<0.001	
Pain acceptance				−0.47^***^
Cognitive fusion				0.01
Decentering				0.05
Committed action				−0.03

*** *p ≤ 0.001*,

** *p ≤ 0.01*,

^*^p < 0.05.

For the second set of analyses, change in sleep interference was the dependent variable. Change in pain was entered in the first step of this analysis, and significantly contributed 15% of the variance to change in sleep interference. Changes in psychological flexibility processes were entered in the second step and together contributed an additional 5% of the variance to change in sleep interference, above and beyond that accounted for by changes in pain. Examination of the beta weights from the final regression equation indicated that changes in pain intensity, β = 0.31, *t*_(240)_ = 5.15, *p* < 0.001, and pain acceptance, β = −0.17, *t*_(240)_ = −2.50, *p* < 0.05, each contributed significant unique variance to the prediction of change in sleep interference. For the pre- to follow-up regression analysis, changes in psychological flexibility processes significantly contributed 19% of the variance to changes in sleep interference, beyond the variance accounted for by changes in pain intensity (32%). In the final regression equation for the follow-up analysis, changes in pain intensity, β = 0.48, *t*_(121)_ = 7.24, *p* < 0.001, and pain acceptance, β = −0.47, *t*_(121)_ = −6.39, *p* < 0.001, both contributed significant unique variance to changes in sleep interference.

For the third analysis, change in sleep efficiency was the dependent variable. Change in pain intensity significantly contributed to the prediction of change in sleep efficiency; however, change in pain only accounted for 3% of the variance in this outcome. Changes in psychological flexibility variables did not significantly contribute additional variance to the prediction of change in sleep efficiency above and beyond the variance accounted for by change in pain. Given the non-significant change in sleep efficiency for the pre-treatment follow-up period, regression analyses were not computed for sleep efficiency over the follow-up period.

## Discussion

There is still relatively little known about the best ways to treat sleeping problems in the context of chronic pain. Producing good outcomes for both sets of problems appears particularly difficult. Here we examined the outcomes and process changes obtained in an intensive, interdisciplinary, pain management course based on ACT for chronic pain in adults. First, a high rate of participants in this treatment reported clinically significant insomnia at pre-treatment, 81.3%. Outcomes on standard pain management outcomes were good, including large effects for pain interference and depression at post-treatment and medium effects at follow-up. Insomnia severity, sleep interference, and sleep-efficiency also improved significantly with a medium effect for sleep interference and small effect for the other two a post treatment. A significant reduction in the proportion of participants taking anxiolytic medication, which are often also used for sleeping problems, was observed at post-treatment. At follow-up, however, only small effects for insomnia severity and sleep interference remained. Furthermore, 42.7–84.5% of participants showed clinically meaningful improvements across outcomes at post treatment, while 6.1–19.8% of participants showed clinically meaningful worsening on post treatment outcomes. At follow-up, 28.8–69.0% of participants showed clinically meaningful improvements across treatment outcomes, while 11.1–28.3% of participants appeared to show clinically meaningful worsening.

A secondary aim of this study was to examine changes in ACT process measures and whether these changes correlated with changes in sleep outcome measures. Pain acceptance, cognitive fusion, decentering, and committed action each improved at post-treatment and follow-up with near small to large effect sizes, including somewhat larger effect sizes at follow-up for pain acceptance and cognitive fusion. Moreover, pre- to post treatment changes in pain acceptance, cognitive fusion, decentering, and committed action correlated in the expected therapeutic direction with improvements in insomnia severity and sleep interference. Only pre- to post-treatment changes in cognitive fusion, decentering, and committed action correlated with improved sleep efficiency and these correlations were quite small. A similar pattern of results as those at post treatment were obtained in the follow-up analyses for correlations between changes in psychological flexibility process variables and improvements in insomnia severity and sleep interference. However, only changes in committed action correlated significantly with sleep efficiency changes, which is perhaps unsurprising given that there was no longer a significant improvement in this variable at follow-up. Taken together, the outcome and process evidence suggest that an ACT-based treatment, even one with minimal sleep treatment content, is associated with improvements in sleep for people with chronic pain, and it appears that improvements in the specific processes targeted within this therapy are related to improvements in treatment outcomes.

The rate for screening positive for possible clinically significant insomnia here is very high and similar to the rate found in previous specialty treatment contexts in the UK where a rate of 79% was found (McCracken et al., 2011). Given the known adverse health impacts of poor sleep (Tang et al., 2007) these rates are startling. While we demonstrate significant effects on sleep outcomes here, the fact that the positive screening rate of insomnia only reduced from 81.3% at pre-treatment to 72.7% at follow-up indicates the need for further treatment developments. This partial recovery rate is similar to the results of Currie et al. (2002). Unlike previous studies of treatments explicitly focused on chronic pain and fatigue (Vitiello et al., 2009; Pigeon et al., 2012) the current treatment appeared to successfully address both pain and sleep-related outcomes, although it may have addressed the pain-related outcomes more successfully.

The improvements in general clinical outcomes and process changes observed here are consistent with those produced in previous studies of ACT for chronic pain (Wicksell et al., 2008; McCracken and Gutiérrez-Martínez, 2011; Wetherell et al., 2011; Trompetter et al., 2015; see Hann and McCracken, 2014 and A-Tjak et al., 2015 for reviews). The support found here for the role of psychological flexibility facets in relation to sleep also is consistent with previous findings (McCracken et al., 2011; Bothelius et al., 2015), particularly the finding that these facets correlate more highly with ratings of sleep quality than directly measured sleep efficiency. In multiple regression analyses of changes in outcomes and process measures, pain acceptance appeared as the strongest unique predictor of sleep outcome change. This may mean that the role of pain acceptance is more important compared to the other process changes when it comes to generating improvements in sleep. Previous studies of ACT for chronic pain also reflect this pattern (Vowles and McCracken, 2008). An alternative explanation is that we are generating smaller effects on the other process measures and this places a ceiling on their apparent role in the regression analyses. Of all of the current facets of psychological flexibility now available to target, acceptance likely is the most familiar and perhaps easiest to engage in an interdisciplinary treatment context. We suggest that our ability to address the other facets, such as cognitive fusion/defusion and committed action, requires greater focus, refinement, and empirical investigation.

It is increasingly recognized that better interdisciplinary treatments for chronic pain will not come from treatment packages that address a little bit of everything, but rather a greater focus and impact on a few key processes and outcome domains, and perhaps from better matching of patients to specific, customized treatments (Williams et al., 2012; McCracken and Morley, 2014). Although the results of the present study seem promising, the outcomes could be better. We suggest that ways to improve upon the results include the following: (a) selection of people with significant insomnia, (b) a greater focus on processes of change that show a significant role in sleep improvement, and (c) a greater targeting of these processes specifically to sleep-related behavior patterns. The issues of greater focus or targeting could mean increased time, intensity, or dosage. It has been suggested that combining psychological flexibility and conventional sleep improvement methods, such as sleep compression, may represent a particularly potent way to address sleep problems (Lundh, 2011; McCracken et al., 2011). To capitalize on this synergy, a more structured and intensive application of the sleep methods would be needed than was done here. Finally, we do not know the full potential role of cognitive defusion and committed action processes in sleep. Greater therapeutic impact on these may yield a larger impact on sleep, but this remains to be further studied.

Another relevant point is that the analyses here are based on group data. When a more specific process like acceptance of pain appears more important than a more general process like committed action or defusion, for example, this does not mean that this applies to every individual. Instead this arises as a pattern in the group. We assume that the barriers for sleep, or skills and capacities to achieve good sleep, are somewhat different for each individual. Thus, it may be useful for future research to examine subgroups for which more specific processes like pain acceptance play a more important role in sleep outcomes than the more general “open, aware, and engaged” processes of psychological flexibility, and vice versa. Greater tailoring of treatment to the specific and general barriers to sleep on a more individualized basis may enhance the treatment effects seen here. In general, we recommend that more “single-subject” research to understand processes of change (e.g., Villatte et al., 2016) combined with user-involvement in method design may improve on the treatment methods and therefore the results here.

There are limitations in the current study. It is not a controlled trial. The study shows changes over time in sleep outcomes and process measures, but we cannot definitively say that ACT produced these improvements in sleep via increased psychological flexibility. It is possible that other components of the treatment contributed to the changes observed. A randomized controlled trial (RCT) design and formal mediation analyses are required to more definitively test this question. Furthermore, clinician competency, treatment and protocol fidelity were not formally monitored, as this study was conducted in routine clinical care and not as a part of a funded RCT, nor was participant session attendance recorded. Within the subsample of participants we analyzed at follow-up, there was considerable attrition. We also know the follow-up completers differed from non-completers in that they reported greater pain acceptance and committed action at post treatment. Therefore, we cannot rule out some biasing effect on the follow-up data. Further, the sample here is highly selected, appearing as they do in a specialty service in central London. Future research is needed to examine the generalizability of the current results to patients with characteristics that differ from the current sample. Finally, the sleep measures used here are retrospective and indirect, and this allows for the influence of recall bias and other sources of inaccuracy. Certainly in the future sleep diaries and perhaps automatic monitoring could improve the quality of data.

In summary, a convincing pattern of significantly disturbed sleep appears in around 8 out of 10 of adult participants in specialty treatment for chronic pain. An intensive, interdisciplinary, ACT-based treatment course with minimal methods to address disturbed sleep here was associated with decreased insomnia severity and interference with sleep both immediately post-treatment and at a 9-month follow-up. Facets of psychological flexibility also improved during this treatment and changes in these were correlated with improvements in sleep, with pain acceptance appearing to play a relatively larger role. There appears to be an opportunity here to follow-up from these results with both a greater focus on sleep methods and a more intensive focus on psychological flexibility to improve sleep outcomes even further, particularly in RCT designs.

## Author contributions

All authors helped to conceive and plan the study and prepared and approved the final manuscript. AD conducted the data collection and analyses, the latter supervised by WS. MH devised and conducted the treatment methods related to sleep. TN conducted preliminary analyses of the data and produced the first draft for parts of the final manuscript. LM supervised the research and treatment, formalized the rationale for the study, and led on the interpretation of findings.

## Funding

Open access for this article was funded by King's College London.

### Conflict of interest statement

The authors declare that the research was conducted in the absence of any commercial or financial relationships that could be construed as a potential conflict of interest.
